# Pilot Study: Effect of Morton’s Extension on the Subtalar Joint Forces in Subjects with Excessive Foot Pronation

**DOI:** 10.3390/s23052505

**Published:** 2023-02-24

**Authors:** Inmaculada C. Palomo-Toucedo, María Luisa González-Elena, Patricia Balestra-Romero, María del Carmen Vázquez-Bautista, Aurora Castro-Méndez, María Reina-Bueno

**Affiliations:** 1 Podiatry Department, University of Seville, 41009 Seville, Spain; 2 Instituto Salud y Deporte Dr. Gallardo, 41009 Seville, Spain

**Keywords:** subtalar joint, force, pronation, supination, Hallux, insole, gait

## Abstract

This study focuses on the assessment of the mechanical effect produced by Morton’s extension as an orthopedic intervention in patients with bilateral foot pronation posture, through a variation in hindfoot and forefoot prone-supinator forces during the stance phase of gait. A quasi-experimental and transversal research was designed comparing three conditions: barefoot (A); wearing footwear with a 3 mm EVA flat insole (B); and wearing a 3 mm EVA flat insole with a 3 mm thick Morton’s extension (C), with respect to the force or time relational to the maximum time of supination or pronation of the subtalar joint (STJ) using a Bertec force plate. Morton’s extension did not show significant differences in the moment during the gait phase in which the maximum pronation force of the STJ is produced, nor in the magnitude of the force, although it decreased. The maximum force of supination increased significantly and was advanced in time. The use of Morton’s extension seems to decrease the maximum force of pronation and increase supination of the subtalar joint. As such, it could be used to improve the biomechanical effects of foot orthoses to control excessive pronation.

## 1. Introduction

Foot pronation is defined as a combined movement of eversion of the calcaneus, abduction and dorsiflexion of the forefoot, being the natural adaptive movement in the subtalar and midtarsal joints for shock absorption in gait [1]. Excessive or long-term, prolonged pronation of the foot could be related to disorders in the lower extremities produced by mechanical adaptation from distal to proximal structures. Additionally, predisposition to overuse pathology affecting the lower back, hip, knee, ankle and foot, in a descending chain, may be identified [2].

In addition, these patients frequently present a first-ray dorsiflexion as the amplitude of movement is higher in the pronation position. This circumstance has a negative impact on the first metatarsophalangeal joint function because the range of extension of the Hallux is reduced, which may produce a Hallux Limitus [3].

Orthopedic interventions affect the kinematics of the hindfoot. Foot orthoses or standard insoles with modifying elements beneath forefoot have been extended used to treat overuse injuries. However, the effect that they produce on the variables of plantar forces and pressures has not been verified [4].

Morton’s extension is an element placed beneath the anterior part of the foot orthosis under the first ray. The length extends from the first metatarsal head to the proximal phalanx of the Hallux. The main objective of this study is to improve the function of the first ray and increase its load because pronation reduces the mechanical efficiency of digital take-off [5].

New technology tools have been used to capture plantar pressures and the force exerted on the foot during different activities. The force plate is a measurement instrument used to analyze plantar force; it measures the reaction force of the ground that is exerted during the dynamics of stepping in three planes of space (Fx, Fy, Fz) [6].

The interest of this study focuses on the assessment of the mechanical effect produced by Morton’s extension as an orthopedic intervention in patients with bilateral foot pronation posture, through the variation in hindfoot and forefoot prone-supinator forces during the stance phase of gait. 

The aim of this study is to identify if there are any modifications when comparing three patient conditions: barefoot (A); wearing footwear with a 3 mm EVA flat insole (B); and wearing a 3 mm EVA flat insole with a 3 mm thick Morton’s extension (C), with respect to the force or time relational to the maximum time of supination or pronation of the subtalar joint.

## 2. Materials and Methods

### 2.1. Trial Design

A quasi-experimental and transversal study was designed. Three situations were compared in relation to the maximum pronation and supination of the subtalar join, with respect to the maximum force and moment. The three conditions were evaluated using a force plate (FP4060-07, Bertec Columbus, OH, USA). The force plate used consists of strain-gage load transducers that precisely measure six components, namely three orthogonal forces and the moments of each axis. The size was 400 mm width × 600 mm length, and the maximum load was 5000 N and the natural frequencies for each plane were as follows: Fz, 340 Hz; Fx, 500 Hz; and Fy, 550 Hz. It was embedded in the ground and connected by output cables without any signal degradation. The digital output was directly plugged into the computer. Data collection was carried out in the Clinical Area of Podiatry of the University of Seville. Authorization was given by the Podiatric Clinical Area of the University of Seville.

### 2.2. Inclusion/Exclusion Criteria

#### 2.2.1. Inclusion Criteria

The inclusion criteria were as follows: men; between 18 and 65 years of age; with bilateral pronation feet posture (FPI values between +6 and +12); and provided signature of the informed consent form prior to intervention.

#### 2.2.2. Exclusion Criteria

The participants were excluded when they have any of the following conditions: history of neuromuscular or osteoarticular diseases; lower limb dysfunction; foot and ankle deformities; unilateral foot pronation; injuries diagnosed in the last 6 months; and/or previous foot surgery. A clinical evaluation of all the participants was previously assessed to verify the symmetry of the legs, and later they were only included when bilateral symmetric pronation of the feet was presented.

#### 2.2.3. Procedure

After verbally informing all subjects who volunteered and met the inclusion criteria to participate in the study of the procedure, they were given an informative document on the nature of the study and an informed consent form.

Patient affiliation data were recorded, including name and date of birth. Later, they were explored and evaluated using the foot posture index (FPI-6) for both feet. The foot posture index is a validated method used to quantify standing foot morphology. There are six criteria to evaluate: (1) talar head palpation; (2) supra and infra lateral malleoli curvature; (3) calcaneal frontal plane position; (4) prominence in the region of the talar neutral joint; (5) congruence of the medial longitudinal arch; and (6) abduction/adduction of the forefoot on the rearfoot. Each item was assessed from −2 to +2, with the range being from −12 to +12. The standing relaxed foot position is considered to be supinated when the total score is from −12 to −1, the neutral posture is from 0 to +5, and the pronated foot posture is from +6 to +12 [7]. Weight was measured in kilograms (kg) through the force given by the force platform in the vertical axis or Z, Fz, with the individual standing on the platform with his normal base of support.

**Figure 1:**
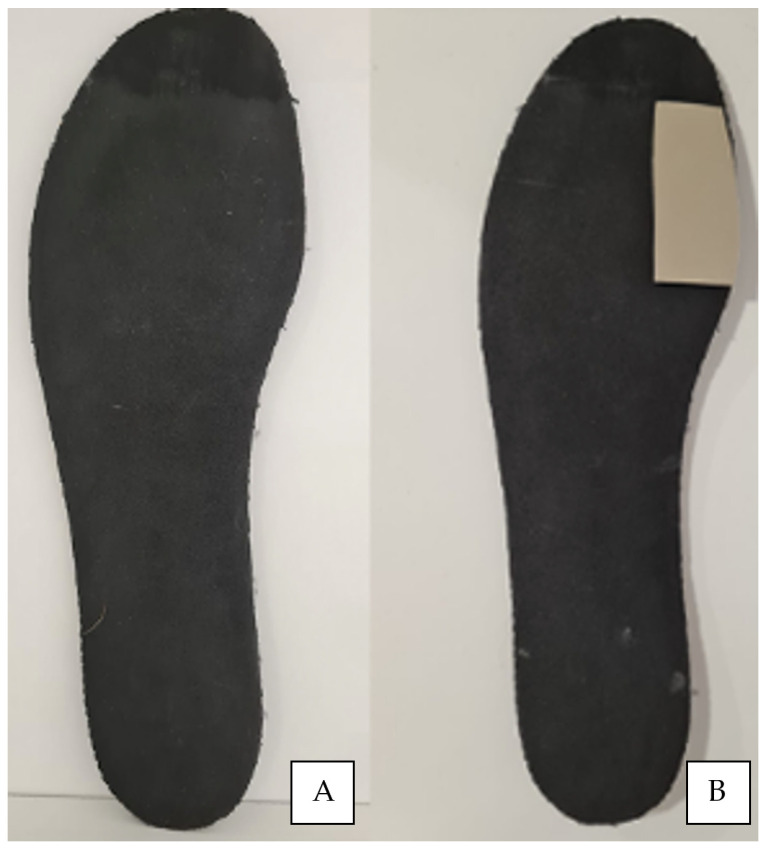
Flat insole (**A**) and flat insole with Morton’s extension beneath first-ray load from a plantar vision (**B**).

Three conditions were studied as independent variables: barefoot (A); wearing footwear with a 3 mm EVA 40–45 shore A flat insole inside (B); and wearing footwear with a 3 mm EVA 40–45 shore A flat insole and with a 3 mm thick Morton’s extension (C) (Figure 1). Due to bilateral pronation, the right foot of each subject was studied.

**Figure 2:**
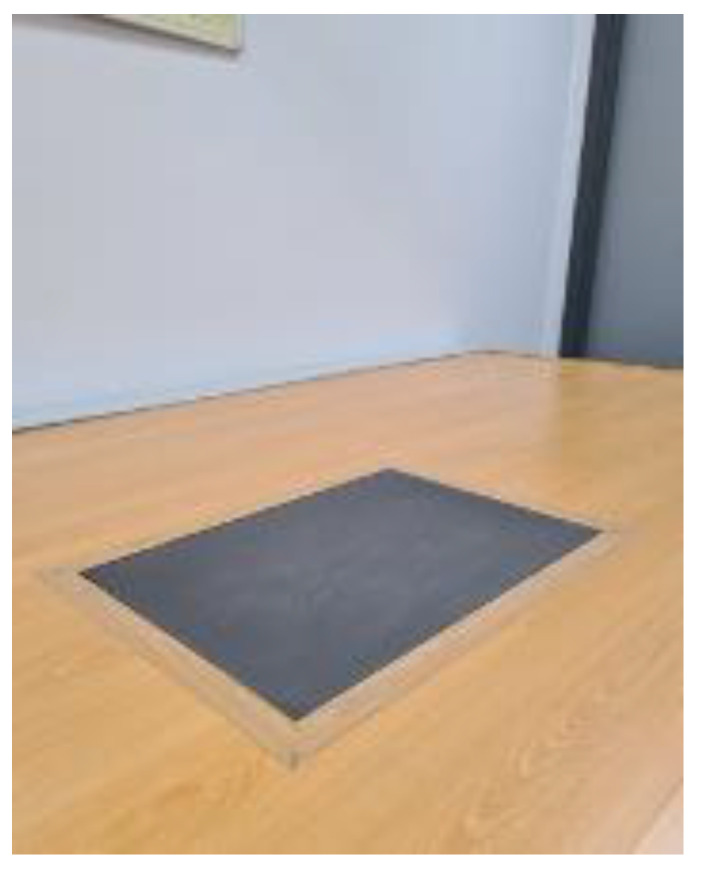
Force platform FP4060-07, Bertec Columbus, OH, USA.

All participants had to walk at their most comfortable speed and always in the same direction. Each of the conditions was recorded 10 times in the lower right limb and at the same velocity on a force platform (FP4060-07, Bertec Columbus, OH, USA, Figure 2), following the same sequence (the mean of the 10 times was used for statistical analyses). There was an acclimation period of 3 min for each of the different conditions. In order to control possible interferences generated by different shoes, all the participants used the same footwear type provided by the research team.

A Microsoft Excel macro was used to treat the force plate data. As the gait cycle has three periods, namely contact (0 to 28%), the period of medium support (28 to 66%) and the propulsion period (67 to 100%), to analyze the whole step, we considered the starting point (heel contact) when the first score was the last 0. Subsequently, the values were recorded until they ended with the last number before the residual zero. The macro identified the values during the stance phase of each step according time, given in N and every 2% of the stance phase duration. For each of the three conditions, maximal pronation forces, maximal supination force, maximal pronation time and maximal supination time were recorded.

The analysis of the data was carried out using statistical software SPSS^®^ version 22.0 for Windows. For the comparison of numerical variables of repeated measures in more than two positions, analysis of variance for repeated means, or the Friedman test, according to the application criteria, was carried out. In case that this test was significant, multiple comparison tests were carried out to detect in which positions the differences were found. In all the hypothesis tests, a significance level of 0.05 was considered.

#### 2.2.4. Ethical Statements

The study was conducted in accordance with the Helsinki Declaration and approved by the Institutional Review Board Committee of the Virgen Macarena and Virgen del Rocío University Hospitals of Seville (Project code: 0973-N-20). This research report takes into account the CONSORT 2010 statement guidelines for randomized clinical trials.

## 3. Results

### 3.1. Description of the Total Sample

The size of the final sample was 15 participants of male sex. The mean age was 39 ± 12 years old (range 22–63) and the mean body weight was 75.8 ± 9.5 kg (median ± SD).

#### 3.1.1. Descriptive Data

Analysis was performed for the maximum pronation and supination forces of the variables under the following different conditions: barefoot (A); wearing footwear with a 3 mm EVA 40 shore A flat insole (B); and wearing footwear with a 3 mm EVA 40 shore A flat insole with a 3 mm thick Morton’s extension (C) (Table 1), corresponding to the mediolateral forces on the X axis, Fx.

When the inferential analysis was performed to compare the different situations with respect to the maximum pronation forces, the results showed that there was no statistical significance (*p* = 0.065). With respect to the maximum supination force, the data showed that there was a statistically significant relationship between the A and B condition (*p* = 0.009) and the A and C condition (*p* = 0.009). 

#### 3.1.2. Interferential Data

The descriptive data with respect to the maximum pronation and supination moments during the gait cycle are shown in Table 2. The comparison of the related quantitative variables was performed between the three conditions, A, B and C (expressed with respect to the total gait cycle duration in percent). The data are presented in Table 2.

After the data comparison of the different maximum pronation moments, the data showed that there is no statistical significance with respect to the three conditions (*p* = 0.712). Different results were found when the maximum supination moments were compared, and a *p* value of 0.001 was observed between the A and B (*p* = 0,002), and A and C (*p* = 0.008) conditions.

## 4. Discussion

The aim of this preliminary study was to evaluate, in a total sample of 15 men with excessive pronation of the foot, if there is a significant effect on the maximum subtalar joint pronation and supination forces, as well as the moments in which they are produced, comparing the three following conditions: barefoot (A); wearing footwear with a flat insole (B); and wearing a flat insole with a 3 mm thick Morton’s extension, measured by a Bertec force plate.

The results show that there are only statistically significant differences for supination maximum force and in the moment produced (time) (*p* < 0.05).

According to the consulted literature and to the best of the investigators’ knowledge, comparison with other works is difficult given the originality of our research. In general terms, the most widely used sensor for the evaluation of the biomechanics of the foot has been the baropodometric plate. Plantar pressures are analyzed in the static position or dynamic, as well as the trajectory of the center of pressure of the foot (COP), before and after intervention.

Authors such as Zhang et al. [8] (2019) and Kerr et al. [9] (2019) evaluated the influence of the use of forefoot wedges on the prone/supination movement of the subtalar joint in order to identify the correlation with the biomechanical disturbances that the wedges induce to other structures. Wong et al. [10] (2008), Hillstrom et al. [11] (2013) and Buldt et al. [12] (2018) observed that, in subjects with foot pronation, the COP moved medially during the gait cycle. Grady et al. (2002) concluded that the use of plantar elements placed beneath the first ray may increase the external rotation and inversion of the hindfoot and they determined that this fact is positively correlated with greater thickness of the element [13]. Nyska et al. (1995) analyzed the kinematics of lower limb changes after applying wedges beneath the first and fifth rays of the foot. These findings suggest that forefoot orthotic elements are effective by modifying dynamics in runners with excessive foot pronation [14]. Van Gheluwe and Dananberg (2004) analyzed the COP trajectory using an insole plantar pressure measurement system, and any significant differences were registered in the maximum temporary variable values (moments). These results are inconsistent with those of our study as the intervention modified the time of maximum supination (barefoot and flat insole, *p* = 0.002; and flat insole and Morton’s extension conditions, *p* = 0.008) [15].

However, our results are consistent with those of Johanson et al. 1994, who determined, by a computerized video-motion-analysis system, the influence of various conditions on pronosupination movement, namely footwear, footwear with an insole and footwear with several insoles with a modified orthotic post beneath the forefoot or the hindfoot. In patients with forefoot varus, plantar pronation seems to increase. Like our results, the isolated forefoot post did not show a significant reduction in subtalar pronation, although the use of an insole and an insole with forefoot posts affected the eversion movement [16].

On the other hand, Costa et al. (2021) determined that the use of insoles with arch support and different medial wedges in the hindfoot reduced the eversion angle of the ankle. This angle decreased during the stroke in the initial support phase and increased in the propulsion phase with wedges of 6 and 9 degrees of inclination during walking and running [17].

In the same way, Bonifacio (2018) verified how the use of medial wedges significantly controlled ankle eversion and improved patellofemoral pain, using a force system and other instruments [18].

In addition, McMillan and Payne 2008 concluded that maximum supination time decreased when orthotic elements were used to control pronation. In our study, we found such a tendency, although there was no statistically significant difference. This could be explained by the fact that the interventions were not isolated in the forefoot, as well as by the small sample size [19].

Mündermann et al. (2003) observed that different orthopedic interventions reduced maximum eversion, without affecting maximum inversion, when analyzing kinematics and kinetics, during running. On the contrary, our findings didn’t show change in maximum supination between the different interventions, except if we compared them with the barefoot condition [20].

Morley et al. (2010) observed that there were relevant differences in terms of the maximum pronation time, occurring before in barefoot conditions and later, in shoe-wearing conditions. In our study, the differences were not significant. The discrepancy could be explained because we only studied participants with excessive foot pronation using a force plate as the measurement instrument [21].

Zhao et al. (2021) explored the effect of arch-support functional insoles. A force plate was used to record the vertical ground reaction force. The first metatarsal region’s contact area was increased and the peak pressure and time–pressure integral of the fist metatarsal [22].

Lourenço et al. (2022) observed the effects of prefabricated and customized insoles on walking, stepping up and stepping down tasks in individuals with excessive foot pronation. The kinetic data were recorded using three synchronized force plates. Both insoles reduced rearfoot eversion in all tasks [23].

Zhang et al. (2022) determined the effects of foot orthoses with forefoot wedge and arch-support components during running in symptomatic pronated feet. The medial forefoot wedge can shift the center of pressure laterally during the midstance and propulsion phases of running. Arch support mainly influenced loading on the medial heel and thereby the center of pressure trajectory in the loading stance phase [24].

Limitations: In our opinion, the main limitation of our study is the small simple with only men as participants, which implies a population bias. Further research could analyze both limbs comparing laterality. In addition, Morton’s extension, as an orthotic intervention, could be determined over time using a longitudinal design study.

## 5. Conclusions

The use of Morton’s extension modifies the maximum force of pronation of the STJ without statistical significance, but the reduction could be clinically relevant. Using this orthotic element, the maximum force of supination increases, and it occurs before the stance phase of the gait.

Morton’s extension could be used to improve the mechanical effects of foot orthoses in patients with excessive pronation.

## Figures and Tables

**Table 1:** Maximum pronation and supination force with respect to the footwear, the flat insole and the flat insole with a Morton’s extension insole situation is shown. The measures are shown in Newton for a 95% confidence interval; a normal distribution of the variables is presented. (Fx axis).

Total Sample *n* = 15	Minimum	Maximum	X(D.S)
Max. pronation barefoot	48.9	92.3	71.2 (13.9)
Max. pronation flat insole	28.7	82	63.5 (13.8)
Max. pronation Morton’s extension	42.4	79.3	64.1 (11.7)
Max. supination barefoot	−124.9	−5.4	−58.3 (39.2)
Max. supination flat insole	−79.4	−9.5	−36.9 (29.9)
Max. supination Morton’s extension	−55	−15	−37.8 (14.2)

Values are presented as average ± standard deviation (SD).

**Table 2:** The pronation and supination moments with respect to the footwear, the footwear with a flat insole and the footwear with a Morton’s extension situation is shown. The measures are shown in Newton for a 95% confidence interval; a normal distribution of the variables is presented. (Fx axis).

Total Sample *n* = 15	Minimum	Maximum	X(D.S)
Max. Pronation barefoot	14	80	43.6 (25.61)
Max. Pronation flat insole	6	80	43.73 (28.8)
Max. Pronation Morton’s	14	78	45.47 (26.98)
Max. Supination barefoot	4	4	---
Max Supination flat insole	4	18	6.67 (3.34)
Max Supination Morton’s	4	8	5.33 (1.48)

Values are presented as average ± standard deviation (SD).

## Data Availability

Not applicable.
